# Effect of carbamylated erythropoietin on neuronal apoptosis in fetal rats during intrauterine hypoxic-ischemic encephalopathy

**DOI:** 10.1186/s40659-019-0234-7

**Published:** 2019-05-13

**Authors:** Min Diao, Yi Qu, Hui Liu, Yushan Ma, Xuemei Lin

**Affiliations:** 1 Department of Anesthesiology, West China Second University Hospital, Sichuan University, No. 20, Section 3, South of Renmin Road, Chengdu, Sichuan China; 2 Key Laboratory of Birth Defects and Related Diseases of Women and Children, Sichuan University, Ministry of Education, Chengdu, Sichuan China

**Keywords:** Carbamylated erythropoietin, Neuronal apoptosis, Intrauterine hypoxic-ischemic encephalopathy

## Abstract

**Background:**

Hypoxic-ischemic encephalopathy (HIE) is a common disease that occurs during the perinatal period. The primary cause of neonatal HIE is related to fetal intrauterine anoxia. Carbamylated erythropoietin (CEPO), a derivative of erythropoietin (EPO), does not exert any erythropoietic effect; however, the neuroprotective effects resemble those of EPO. Previous studies have shown the potential benefits of CEPO on the central nervous system. The present study aimed to investigate the role of CEPO in neuronal apoptosis during intrauterine HIE and the underlying mechanisms.

**Results:**

To validate our hypothesis, we established an intrauterine HIE model by occluding the bilateral utero-ovarian arteries of pregnant Sprague–Dawley rats. Compared to the I/R group, neuronal apoptosis in the CEPO group was significantly lower at 4, 12, 24, and 48 h (P < 0.05). CEPO significantly inhibited CC3 expression (P < 0.05) during the early-stages after ischemia–reperfusion (0.5, 4, 8, 12 and 24 h), upregulated Bcl-2 expression, and downregulated Bax expression at 4, 8, 12, and 24 h (P < 0.05).

**Conclusions:**

Carbamylated erythropoietin pretreatment inhibited the expression of proapoptotic protein CC3 in brain and regulated the Bcl-2/Bax ratio, resulting in reduced neuronal apoptosis and thus resulting in a protective effect on intrauterine HIE.

## Background

Hypoxic-ischemic encephalopathy (HIE) is one of the major causes of death and disability in children, and is primarily induced by fetal distress and intrapartum asphyxia [1]. HIE not only affects the child’s intellectual level but may also lead to severe neurological dysfunction, as well as perinatal death [2]. Of the 2.8 million neonatal deaths in 2013, 0.64 million deaths (22.9%) were estimated to be caused by intrapartum asphyxia [3]. In recent years, obstetric and neonatal medicine has made progress to improve HIE prognosis; however, effective prophylaxis and treatments are yet lacking.

Erythropoietin (EPO) is the main cytokine involved in erythropoiesis in vivo, and inhibits apoptosis of erythroid progenitors in the bone marrow [4]. Recombinant human erythropoietin (rhEPO), synthesized using recombinant DNA technology, has been widely used in the clinical treatment of anemia caused by various diseases such as cancer [5], premature labor [6], and chronic kidney disease [7]. Recently, studies have shown that EPO exerts a hematopoietic as well as a protective effect on tissues, especially in the central nervous system (CNS).

Erythropoietin and erythropoietin receptor (EPOR) expression in the CNS is significantly increased after ischemia, hypoxia, and other stress conditions, suggesting that EPO might be an endogenous neuroprotective protein [8]. However, endogenous EPO production is limited, indicating that it might be effectively supplied to protect the brain against HIE. In previous studies, we demonstrated that EPO could antagonize intrauterine HIE through the placenta and blood–brain barrier (BBB) [9, 10]. However, high-doses or long-term exposure to EPO may cause an over-stimulation in the bone marrow hematopoietic system leading to polycythemia, hypertension, and thrombosis [11]. This phenomenon is observed especially during pregnancy in the hypercoagulable state in patients, wherein EPO increases the perinatal risk of thrombosis [12], limiting the obstetric applications.

Carbamylated erythropoietin (CEPO), a homolog of EPO, does not exert any erythropoietic effects but is similar to EPO with respect to stability, BBB permeability, and pharmacokinetics [13]. The neuroprotective effects of CEPO resemble that of EPO in cerebral infarction [14], traumatic brain injury [15], experimental autoimmune encephalomyelitis [16], diabetic neuropathy [17], and other models without hematopoietic activity [13]. However, whether administration of CEPO on pregnant mothers exerts a protective effect against fetal neurological damage in the intrauterine hypoxic-ischemic state has not been reported. Hence, in this study, we established an intrauterine HI model using fetal rats to investigate the neuroprotective effects of CEPO on neuronal apoptosis and the underlying mechanisms.

## Results

### Identification of rhEPO carbamylation reaction

**Fig. 1:**
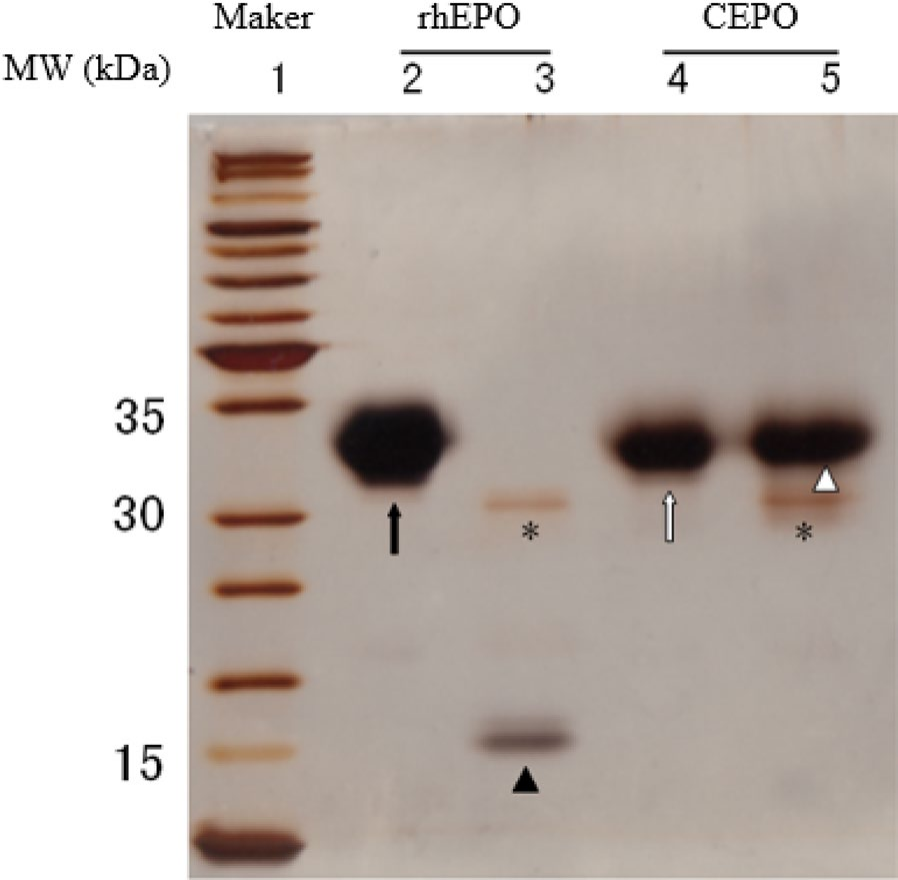
CEPO identification using SDS-PAGE electrophoresis. Lane 1: markers; Lane 2: rhEPO, a broad band of 34 kDa (black arrow); Lane 3: rhEPO + endoproteinase Lys-C, 31 kDa (asterisk) and 16 kDa (filled triangle); Lane 4: CEPO, band around 34 kDa (white arrow); Lane 5: CEPO + endoproteinase Lys-C, a broad band around 34 kDa (white triangle) and a thinner band near 31KDA (asterisk). The 31 kDa band present in all lanes may be due to the Lys-C protein

Endoproteinase carboxyl side of lysine (Lys-C) breaks the peptide chains of lysine [18]. The molecular weight of rhEPO is about 34 kDa and, the cleaved chains are approximately 16 kDa. Lysine residues, converted to homocitrulline by carbamylation, cannot be cleaved by endoproteinase Lys-C. Therefore, the CEPO protein chain will remain about 34 kDa. As shown in Fig. 1, the 2nd and 4th lanes are rhEPO and CEPO with a molecular mass of 34 kDa; the 3rd and 5th lanes are reaction products with endoproteinase Lys-C. In the 3rd lane, rhEPO reacted with the endoproteinase Lys-C and the chains were cleaved with a molecular mass of 16 kDa; in the 5th lane, CEPO was not cleaved by endoproteinase Lys-C, and hence, only 34 kDa but not 16 kDa peptides were observed, indicating that EPO was completely converted to CEPO according to our synthesis protocol.

### Birth state and death rate of fetal rats for each group

In the sham group, pups had ruddy skin and satisfactory limb movements but no fetal death. In the hypoxic-ischemic reperfusion (I/R) group, body appearances did not show obvious abnormalities at 0.5 h. However, at 4 h, the fetuses appeared violaceous, reflexes were weak and some fetal death occurred. The mortality was increased at 24 h. CEPO pretreatment improved the fetal situation but did not alter neonatal mortality. Compared to the I/R group, a significant difference was observed only at 48 h (Table 1, P = 0.045).

**Table 1:** Death rate of rat fetuses in the I/R and CEPO + I/R groups

Time (h)	I/R		CEPO + I/R		P
	Total	Dead (%)	Total	Dead (%)	
0.5	33	2 (6.06)	36	1 (2.78)	0.603
4	43	5 (11.63)	32	0 (0)	0.067
8	36	4 (11.11)	44	4 (9.09)	1
12	42	3 (7.14)	33	2 (6.06)	1
24	55	7 (12.73)	50	6 (12)	0.762
48	43	8 (18.6)	47	2 (4.26)	0.045*

*P < 0.05 compared to I/R group, Fisher’s exact test

### CEPO alleviated brain morphology changes

**Fig. 2:**
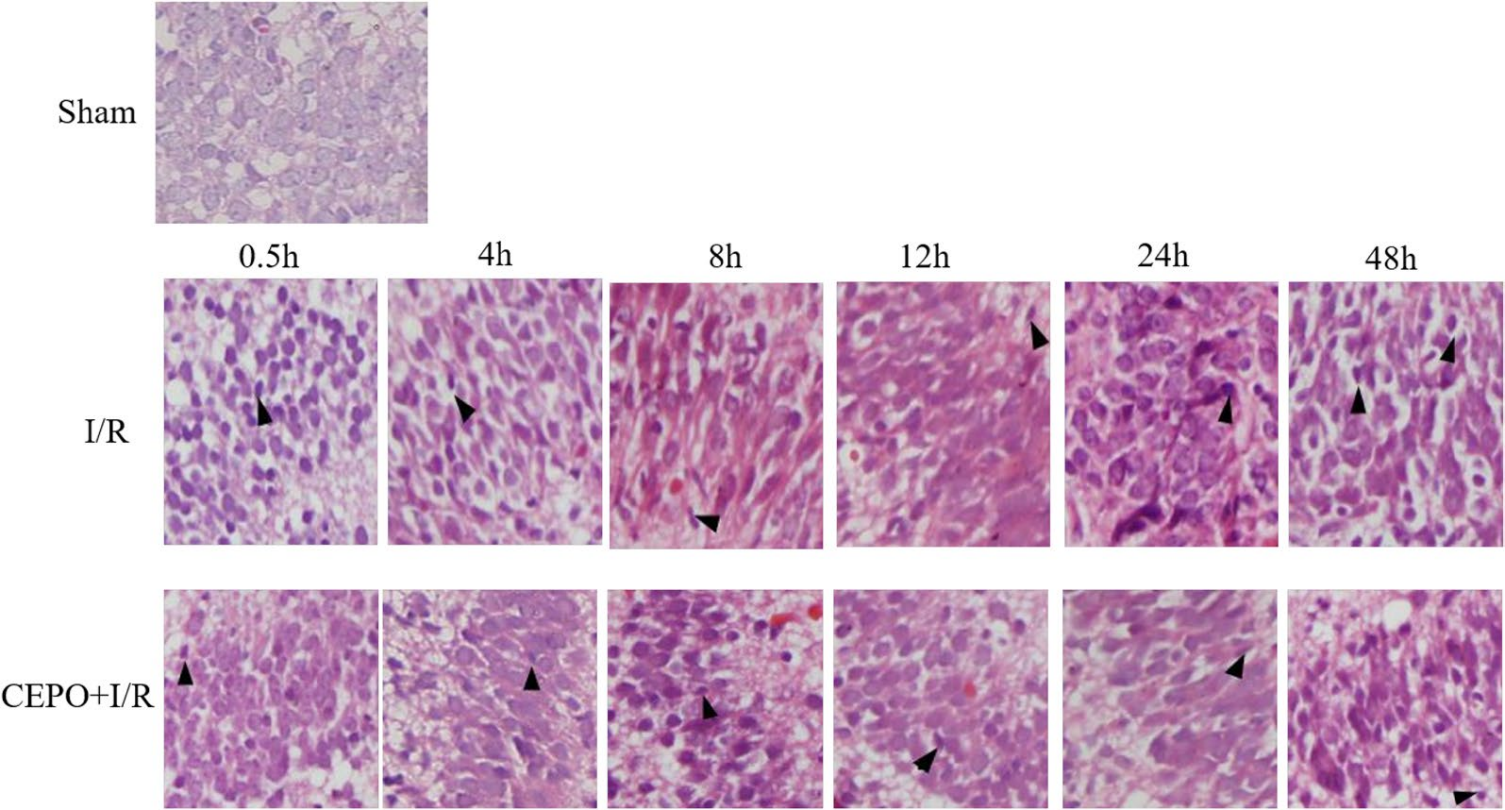
Evaluations of HE staining of rats at different time point in groups. Photomicrographs show morphological changes stained with H&E in brain section (× 400). Karyopyknosis is shown by filled triangle

Hematoxylin–eosin (HE) staining demonstrated that intrauterine ischemia–hypoxia (HI) induced pathological changes in the brain cells. In the sham group, the morphology of the brain was normal and neural degeneration was rarely observed. I/R group showed edema in glial cells, disorders in the pyramidal cell arrangement, reduced cell volume, irregular nuclear chromatin, karyopyknosis, and eosinophilic cytoplasm (Fig. 2). Qualitative analyses demonstrated that CEPO preconditioning reduced these pathological changes.

### CEPO administration inhibited apoptosis

**Fig. 3:**
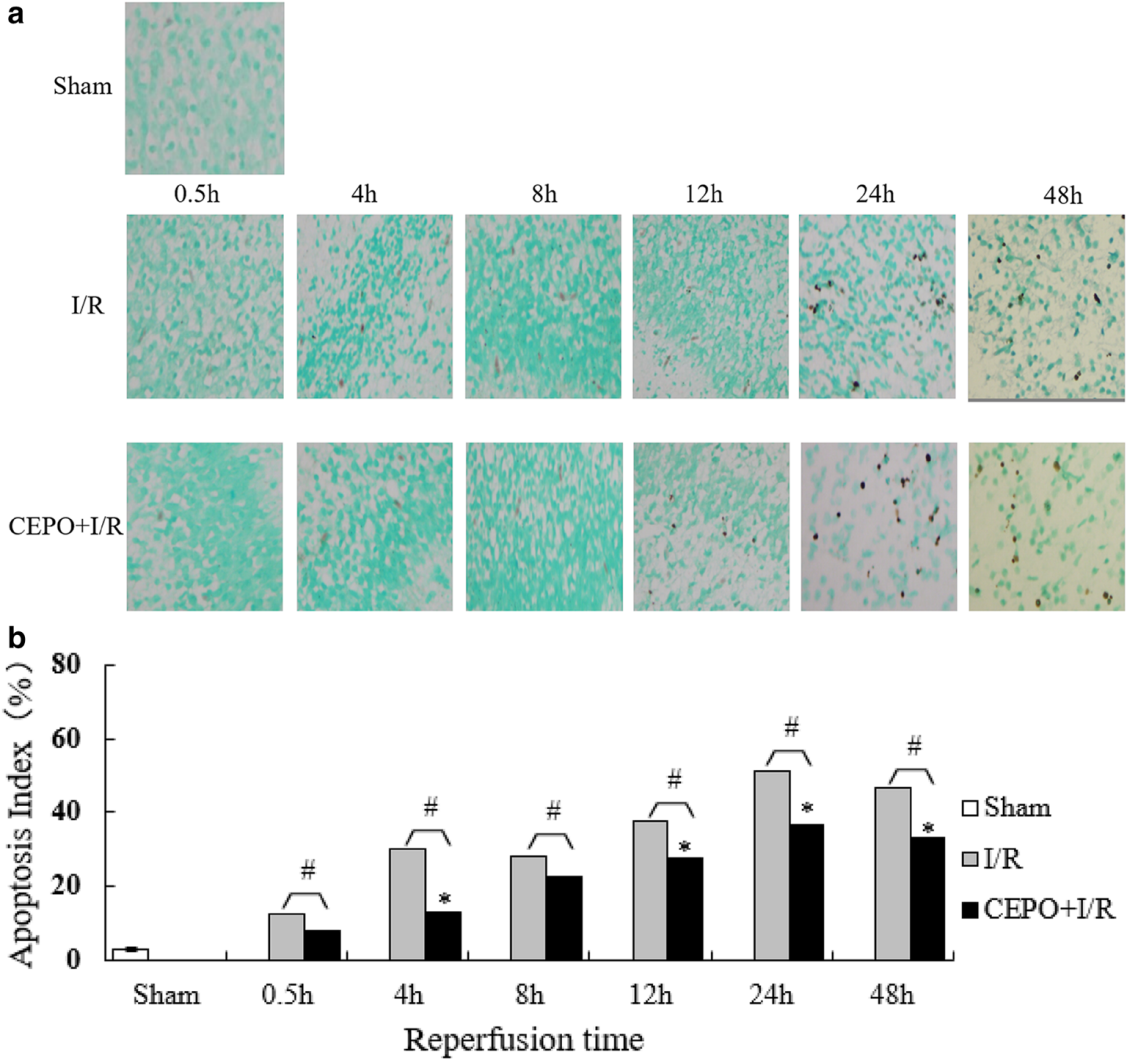
Apoptosis levels of brain cells after reperfusion. **a** Immunoreactive cells after TUNEL staining (400×); **b** statistics of apoptotic indexes (%). The quantitative results are obtained using Image Pro Plus 6.0 image analysis software. Data are presented as mean ± SD of N = 4; *P < 0.05 compared to I/R group, student’s t-test; ^#^P < 0.05 compared to sham group, ANOVA

Terminal deoxynucleotidyl transferase-mediated dUTP nick end-labeling (TUNEL) staining was used to analyze the apoptotic index. The sham group showed 2.67 ± 0.57% positive cells in brain. The apoptotic index of the I/R and CEPO groups at different time points is shown in Fig. 3. Apoptosis increased gradually and reached a peak at 24 h post-reperfusion. Compared to the I/R group, apoptotic indexes for the CEPO group was significantly lower (one-way ANOVA, P < 0.05) at 4, 12, 24, and 48 h. This demonstrated that CEPO inhibited the intrauterine HI-induced neuronal apoptosis effectively.

### CEPO inhibits CC3 expression after reperfusion

**Fig. 4:**
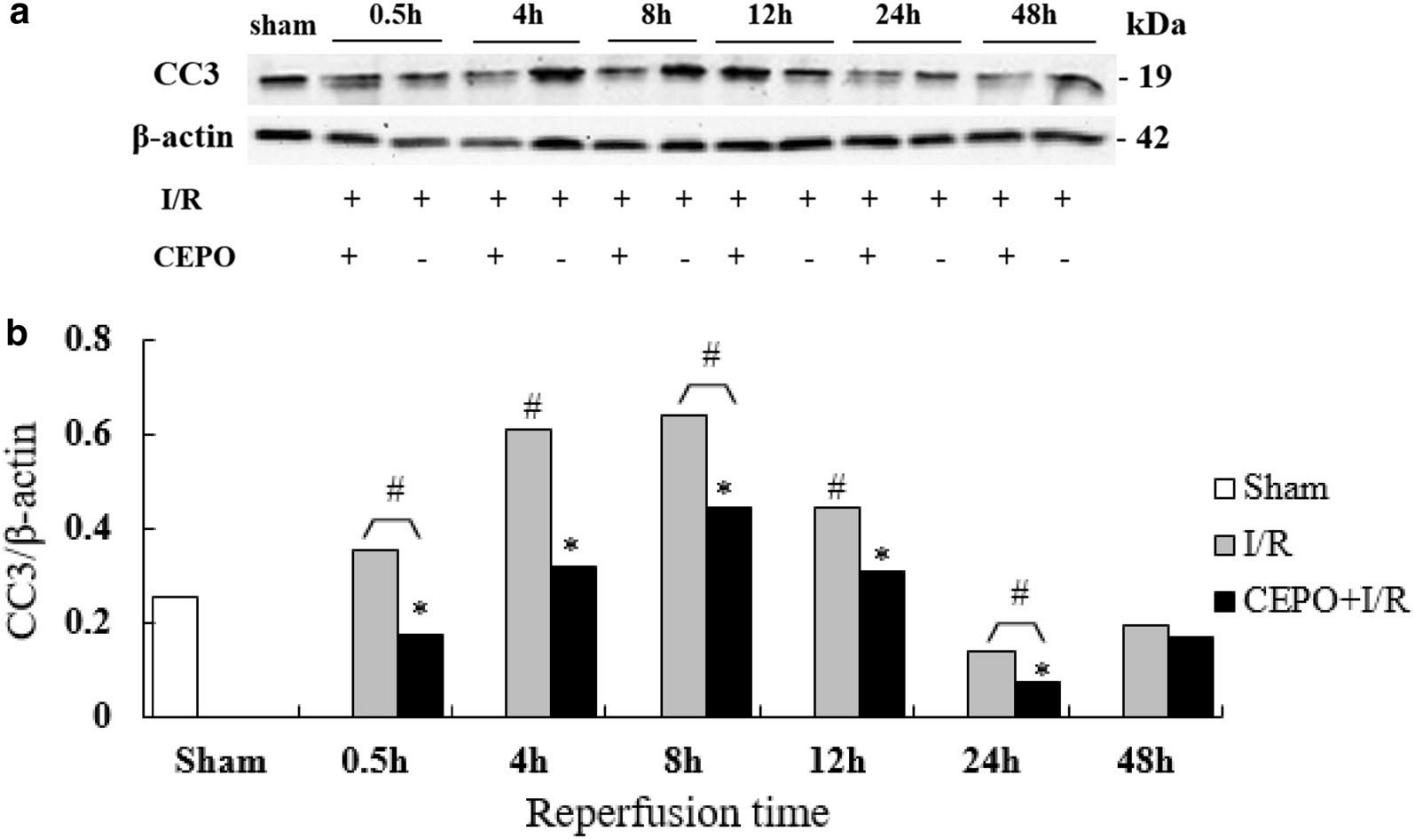
CC3 expression of brain cells after reperfusion. **a** CC3 expression by Western blotting at different time points; **b** relative expression of CC3. The quantitative results are obtained using Image J software. Data are presented as mean ± SD of N = 4; *P < 0.05 compared to I/R group, student’s t-test; ^#^P < 0.05 compared to sham group, ANOVA

The cleaved caspase-3 (CC3) expression increased gradually in the I/R group and peaked levels at 8 h (one-way ANOVA, P < 0.05 vs. sham), followed by a gradual decline (Fig. 4). The CC3 levels in the CEPO + I/R group were significantly lower compared to the I/R group at 0.5, 4, 8, 12 h and 24 h (one-way ANOVA, P < 0.05), which was inconsistent with the levels of apoptosis.

### CEPO pretreatment upregulated Bcl-2 expression and downregulated Bax expression

**Fig. 5:**
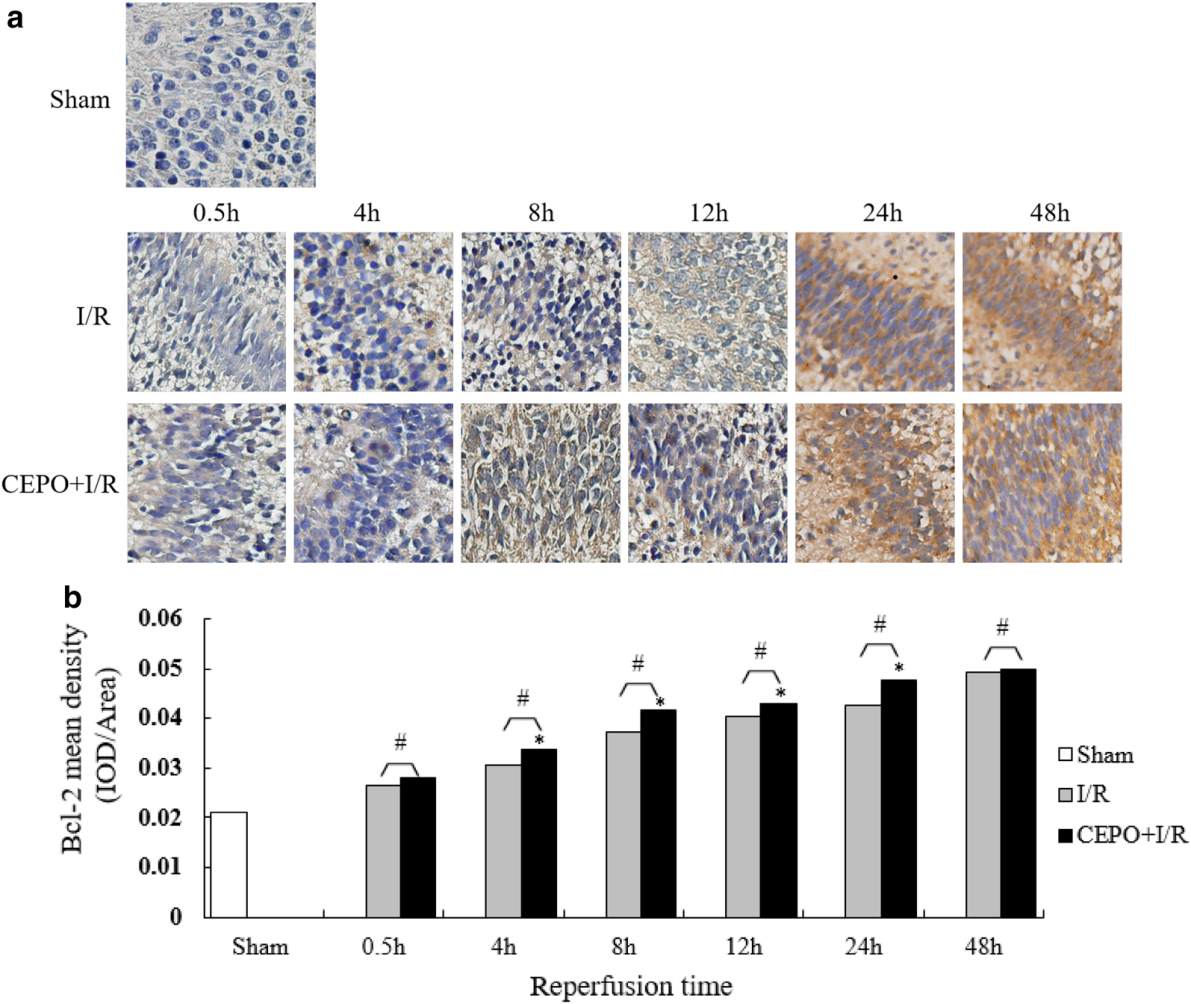
CEPO upregulated Bcl-2 expression of brain cells. **a** Bcl-2 immunoreactive cells at 0.5–48 h time points (400×); **b** quantitative analysis of Bcl-2 protein expression. The quantitative results are obtained using Image Pro Plus 6.0 image analysis software. Data are presented as mean ± SD of N = 4; *P < 0.05 compared to I/R group, student’s t-test; ^#^P < 0.05 compared to sham group, ANOVA

**Fig. 6:**
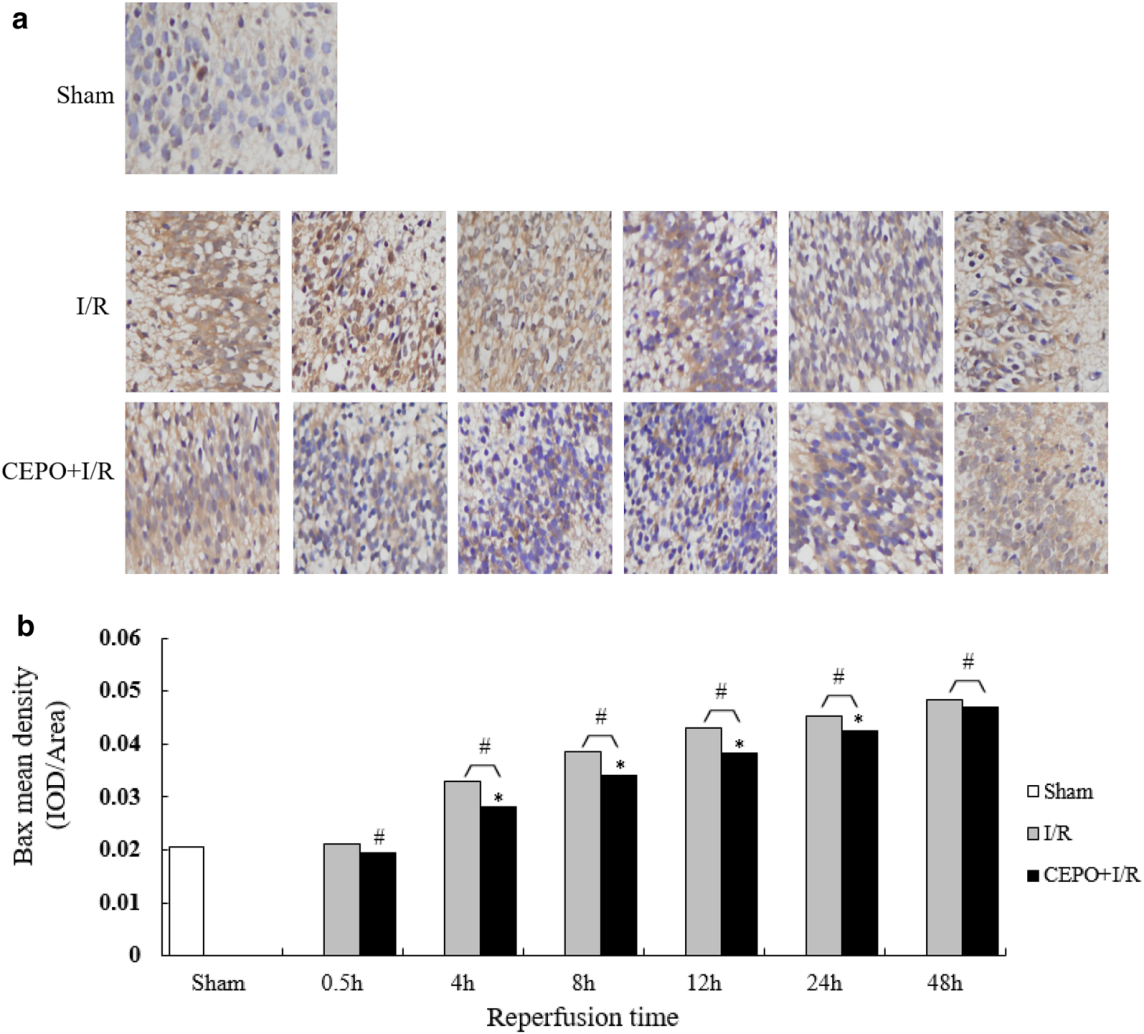
CEPO downregulated Bax expression of brain cells. **a** Bax immunoreactive cells at 0.5–48 h time points (400×); **b** quantitative analysis of Bax protein expression. The quantitative results are obtained using Image Pro Plus 6.0 image analysis software. Data are presented as mean ± SD of N = 4; *P < 0.05 compared to I/R group, student’s t-test; ^#^P < 0.05 compared to sham group, ANOVA

**Fig. 7:**
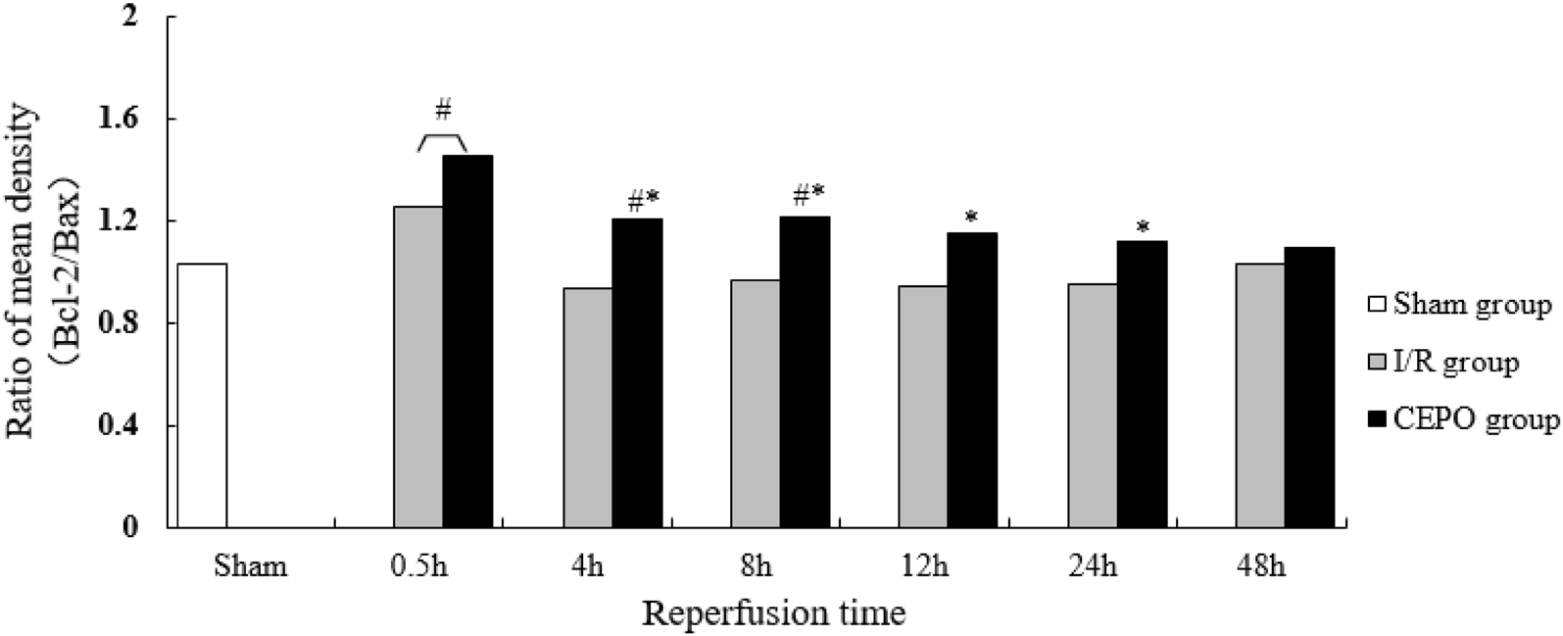
CEPO upregulated Bcl-2/Bax ratio after reperfusion. The relative density of Bcl-2/Bax expression at different time points after reperfusion. Data are presented as mean ± SD of N = 4; *P < 0.05 compared to I/R group, student’s t-test; ^#^P < 0.05 compared to sham group, ANOVA

Immunohistochemistry (IHC) was used to determine the expression levels of B-cell lymphoma 2 (Bcl-2) and Bcl-2 Associated X Protein (Bax) expression levels in the brain cells at different time points after reperfusion. The changes in Bcl-2 and Bax expression are shown in Figs. 5 and 6. Compared to the I/R group, a significant upregulation in Bcl-2 expression was observed in the CEPO + I/R group (P < 0.05) except at 0.5 and 48 h. In addition, Bax expression decreased significantly in the CEPO + I/R group except at 0.5 and 48 h. Furthermore, quantitative analyses demonstrated that CEPO pre-treatment enhanced the I/R-induced Bcl-2 upregulation and suppressed the I/R-induced Bax upregulation. The ratio of relative densities was calculated using Bcl-2/Bax, which indicated anti-apoptotic activity. This ratio was significantly higher in the CEPO + I/R group compared to the I/R group during 4–24 h after reperfusion (Fig. 7, one-way ANOVA, P < 0.05).

## Discussion

The results of the present study demonstrated that a single dose of CEPO administered intraperitoneally in pregnant rats before intrauterine HI insult decreased apoptosis and apoptosis-related proteins in fetal rats. Notably, CEPO pre-treatment could protect fetal rats from intrauterine hypoxic-ischemic brain injury through its anti-apoptotic effect. However, the underlying molecular mechanism needs further investigation.

Previous studies have demonstrated that CEPO, a derivative of EPO, does not stimulate the bone marrow hematopoietic system but has a similar neuroprotective effect as EPO on tissues. This effect has been demonstrated in various diseases such as cerebral infarction [13, 19], spinal cord injury [13], experimental autoimmune encephalomyelitis [16] and chemotherapy-induced toxicities on peripheral nerves [20]. In addition, CEPO promoted neuronal differentiation in neural stem cells [21]. Collectively these studies demonstrated that CEPO is a novel neuroprotective agent with promising clinical applications.

Only few studies have demonstrated that CEPO exerts protective effect against intrauterine HIE. In the current study, healthy pregnant rats were used to establish the intrauterine HIE model. Transient occlusion of bilateral utero-ovarian vessels can cause ischemia–reperfusion, lead to inadequacy of placental blood flow and mimic the sudden interruption block of fetal–placental circulation. Then, we compared the death rates and pathological changes in the hippocampus of fetal rats. CEPO pretreatment significantly reduced the pathological changes and mortality at 48 h compared to the I/R group. TUNEL assays demonstrated that CEPO protected the fetal rat brain from apoptosis by reducing the apoptotic rates. These results were consistent with our previous findings on EPO [9].

Neuronal death after hypoxia–ischemia injury is associated with cell death and apoptosis. In immature hypoxic-ischemic brains, apoptosis plays a crucial role. Different brain regions showed different sensitivities to hypoxic-ischemic stresses. Cerebral cortex and hippocampus were the two most sensitive areas to hypoxia–ischemia injury [22]. In the current study, TUNEL-positive cells were rarely visible in the hippocampus of the sham group. The number of apoptotic neurons in the I/R or CEPO treatment group increased with reperfusion time and reached a peak at 24 h, demonstrating that intrauterine HIE could cause typical neuronal apoptosis in the hippocampus of the fetal rat. Compared to the I/R group, CEPO pretreatment significantly reduced apoptosis in brain cells measured at similar time points. These results suggested that intrauterine HIE was directly associated with apoptosis, and CEPO played a neuroprotective role by regulating apoptosis.

Apoptosis is a complex process, mediated by the caspase family members including the protease cascades. This family encompasses different death-signal transduction proteins, which in turn, induces apoptosis [23]. Among these, caspase-3 is the core member. CC3 is an active form of caspase-3, which is highly expressed during neuronal apoptosis [24]. It is also a critical molecule that mediates cell necroptosis. Furthermore, detecting CC3 expression is a common approach to study apoptosis during brain damage [25, 26]. In the current study, increased CC3 expression was observed with time and reached peak levels at 8 h after reperfusion in the clamping and reperfusion group, followed by a gradual decrease. However, CC3 expression was not consistent with the apoptosis index, which peaked at 24 h. We speculated that the different detection methods resulted in inconsistent data. CC3 expression levels were detected by Western blot on brain tissues preserved in liquid nitrogen, while apoptosis was assessed using TUNEL staining on paraffin-embedded sections. A previous study [25] also reported that inconsistent data was observed when measuring apoptosis and CC3 expression. Nevertheless, the present study showed that CEPO significantly downregulated the CC3 expression at similar time points compared to the I/R group. The effect of CEPO on CC3 was similar to the findings of previous studies using different animal models [27, 28]. These findings suggest that CEPO might sufficiently provide early beneficial effects for intrauterine HIE.

Cell apoptosis is regulated by both pro- and anti-apoptotic proteins. In addition to CC3 expression, the Bcl-2 family is a major regulator of apoptosis. Two proteins in this family (Bcl-2 and Bax) are crucial for the regulation of apoptosis [29, 30]. Bcl-2 is an anti-apoptotic protein that blocks the release of cytochrome C from the mitochondria to the cytoplasm [31]. Bax is a pro-apoptotic member of the Bcl-2 family. Under apoptotic stimulus, Bax translocates from the cytoplasm to the mitochondrial outer membrane, inducing cytochrome C release and caspase-9 activation (by self-cleavage), which results in caspase-3 activation and ultimately apoptosis [32]. Bcl-2 and Bax antagonize each other to determine the cell fate [30]. When the ratio of Bcl-2/Bax is reduced, apoptosis is induced and vice versa when increased [33]. Our results demonstrated that Bcl-2 and Bax levels were increased in fetal rats after intrauterine ischemia and hypoxia, indicating that the expression of protective proteins was initiated along with apoptosis. However, Bcl-2/Bax ratio gradually declined over time, which indicated that cells were still in the apoptotic stage. Compared to the I/R group, Bcl-2 expression in the CEPO group was significantly increased; with a simultaneous increase in Bcl-2/Bax ratios. Taken together, these findings suggested that CEPO regulated neuronal apoptosis by increasing Bcl-2 expression and inhibiting Bax expression. The current results are in agreement with previously published studies [28].

The present study has some limitations. First, we administered a single dose of 50 μg/kg CEPO before intrauterine HI insult. Although not appropriate for potential clinical use, this time point was selected to investigate the protective effects of CEPO in the developing rat brain with HI, as described previously for EPO [9, 10]. In animals with brain injury, CEPO has been shown to be effective within a therapeutic window of at least 6 h [34, 35], and a triple dose of CEPO further reduced lesion volume and improved neurological functional recovery compared to a single dose of CEPO [34]. Whether repeated doses of CEPO or a delayed timing of treatment are equally effective in the developing rat brain with HI is yet to be deciphered. Second, the cellular mechanisms of HI injury demonstrated sexual dimorphism and the male gender was vulnerable to neonatal HIE during the perinatal period [36, 37]. Due to the lack of recognition of sexual dimorphism, we did not verify the pup’s gender during the study. Although the gender of the animals used in the previous study exploring cellular mechanisms of HI [38] was not mentioned, all litters should be gender-typed in the follow-up studies. Third, although the current experimental design had several time points, the association between brain injury and reperfusion time was not explored further. In addition, due to the intraperitoneal maternal body approach, the permeability of CEPO through placenta barrier was not measured in our study. Thus, whether permeability of CEPO was similar to that of EPO is yet to be determined.

## Conclusions

Carbamylated erythropoietin pretreatment inhibited neuronal apoptosis in the hippocampus after intrauterine HI by inhibiting the expression of pro-apoptotic protein CC3 and upregulating the ratio of Bcl-2/Bax, thus inducing a neuroprotective effect. However, the signaling pathways involved in this CEPO neuroprotective effect were not explored in our animal models. To understand the detailed molecular mechanisms, further investigations using in vivo and in vitro studies are needed.

## Methods

### Experimental animals

A total of 52 healthy pregnant Sprague–Dawley (SD) rats (240–260 g) were purchased from Dashuo Experimental Animal Co., Ltd (Chengdu, China). Animals were fed breeding-stage special pelleted fodder at room temperature (22 °C) and 85% humidity. Sichuan University Committee on Animal Research approved all animal studies that were carried out in accordance with the approved guidelines.

### CEPO preparation and identification

Carbamylated erythropoietin was prepared as described previously [13]. Briefly, 1 mL rhEPO (Merck, German) was mixed with 1 mol/L sodium borate (pH 8.8) and 162.22 mg potassium cyanate (KOCN) for a final concentration of KOCN at 1 mol/L. The mixture was incubated in a water bath (37 °C) for 24 h and then transferred into a dialysis bag. The contents were stirred to accelerate dialysis to remove excess KOCN at 4 °C in Milli-Q water followed by dialysis at 4 °C in 1000 mL solution (20 mmol/L sodium citrate and 0.1 mol/L NaCl, pH 6.0). The dialyzed solution was ultrafiltered (Ultrafiltration device, 10 kDa, Millipore) to a concentration > 20 μg/mL. CEPO concentration was measured using an ultramicro-spectrophotometer (Thermo Scientific Nanodrop 2000). Powder was obtained by using the freeze-dry method and stored at − 20 °C for subsequent use. The lysine digestion method [13] was used to confirm whether rhEPO was completely transformed to CEPO. 200 μg powder was solubilized in 200 μL of 6 mol/L guanidine hydrochloride or 250 mmol/L of Tris (pH 9.5). A volume of 25 μL of 0.1 mol/L dithiothreitol (DTE) was added to each solution and then incubated in the dark at 37 °C for 30 min. Then, 25 μL of 0.6 mol/L iodide acetamide was added, followed by incubation at room temperature for 60 min. Finally, 750 μL of 50 mmol/L ammonium bicarbonate and 0.4 mol/L urea were added, and the total volume of 1 mL was dialyzed against 50 mmol/L ammonium bicarbonate and 0.4 mol/L urea solution overnight, during which the dialysate was regularly changed. Concentration was measured using an ultramicro-spectrophotometer (Nanodrop 2000). Concurrently, 20 μg rhEPO or CEPO powder was added to 0.8 μg endoproteinase Lys-C (Calbiochem, Germany), and incubated for 20 h at 37 °C. The digested solution was electrophoresed using a 12% sodium dodecyl sulfate-polyacrylamide gel electrophoresis (SDS-PAGE), and subsequently, the gel was analyzed by silver staining.

### Pregnancy determination

Sprague–Dawley rats that had no prior mating were breed during the estrus phase (female:male = 2:1), and at 9:00 a.m. on the following day, the vaginal swabs were smeared. The detection of sperm in the vaginal smear was defined as pregnancy day 1. Rats were transferred to new cages until pregnancy day 19.

### Intrauterine HI model

Intrauterine HI model was made based on the procedure used in Tanaka et al. [39]. 19-day pregnant rats were anesthetized with 1% pentobarbital sodium intraperitoneally (40 mg/kg, i.p.). A midline incision was performed in the lower abdomen that exposed both the uterine and ovarian arteries. The intrauterine hypoxic-ischemic state was induced using four artery clamps that restricted the bilateral uterine arteries for 30 min. During the clamping process, saline gauzes were frequently changed. The uterus and abdomen were kept warm using a heating lamp. After 30 min, the clamps were removed and the abdomen was sutured, and then transferred back to clean cages.

### Animal grouping and treatment

A total of 52 pregnant SD rats were randomly divided into three groups: the sham operation group (sham, n = 4), the intrauterine hypoxic-ischemic reperfusion group (I/R, n = 4 at each time point, total 24 animals), and the CEPO pretreatment group before initiation of intrauterine hypoxic-ischemic operation (CEPO + I/R, n = 4 at each time point, total 24 animals). The I/R and CEPO + I/R groups were injected with 1 mL normal saline or CEPO (50 µg/kg) through the tail vein 30 min before the operation. The sham group underwent only an abdominal exposure operation without bilateral uterine artery ligation. Similar to the I/R and CEPO + I/R groups, the sham group was injected with 1 mL saline 30 min before the operation, and pups were delivered after 24 h by cesarean section. I/R and CEPO + I/R groups were observed for 6 time points, 0.5, 4, 8, 12, 24, and 48 h after intrauterine hypoxic-ischemic operation. At the corresponding time points above, the uterus horns of pregnant rats were opened, the pups were removed by cesarean section and sacrificed quickly on an ice table to obtain sample of the whole brain tissues for each time point. Some of the brains were frozen in liquid nitrogen, and then, stored at − 80 °C for Western blotting, while others were sliced into 5-μm paraffin-embedded sections for IHC staining, HE staining, and TUNEL staining. Sham group was designed to set only a time point for control compared with I/R group and CEPO + I/R group based on previous studies [25, 26].

### Determining fetal mortality

The number of fetuses from each pregnant rat was recorded at the time of surgery, and the number of dead fetuses from each litter was recorded at the time of cesarean section. Fetal mortality was calculated as the number of dead fetuses divided by the total number of fetuses for each group. Results were expressed as percentages.

### TUNEL staining

TUNEL staining was performed using the FragEL™ DNA Fragmentation Detection Kit, Colorimetric-terminal deoxynucleotidyl transferase (TdT) Enzyme (Merck). Briefly, sections were deparaffinized and permeabilized with 20 µg/mL proteinase K (pH 7.4–8.0) for 20 min at room temperature. Then, the sections were incubated with 3% H_2_O_2_ for 5 min at room temperature, followed by incubation with 1× TdT equilibrium buffer for 10 min at room temperature. After washing, the sections were incubated with TdT enzyme for 60 min at 37 °C. The reaction was terminated by incubation in a blocking/wash buffer for 10 min at room temperature. Subsequently, the sections were incubated with 100 μL of 1× conjugate for 30 min at room temperature and stained with 3,3-diaminobenzidine (DAB) solution in the dark for 10 min. Negative controls without TdT enzyme were also included for each assay. Cells were defined as TUNEL-positive if the nuclei were stained brown. For quantification, the number of TUNEL-positive cells was counted for every 100 cells using five view fields per section at 400× magnification using Image-Pro Plus 6.0 image analysis software. Apoptotic index was calculated as the number of TUNEL-positive cells divided by 100 cells and averaged.

### Western blot analysis

Brain tissue was homogenized in radioimmunoprecipitation (RIPA) lysis buffer supplemented with phenylmethylsulfonylfluoride (PMSF) solution, a protease inhibitor. The homogenates were centrifuged (12,000×*g* for 10 min at 4 °C), and the total protein in the supernatant was measured using the BCA Protein Assay kit (Beyotime Biotechnology). An equivalent of 80 μg protein was analyzed by 15% SDS-PAGE and transferred to polyvinylidene difluoride (PVDF) membrane (Roche). Membranes were blocked with 5% (w/v) bovine serum albumin (Millipore, USA) in 10 mM Tris-buffered saline with 0.1% Tween 20 (TBS-T) for 2 h, and then probed overnight at 4 °C with specific primary antibodies: CC3 (1:500, Cell Signaling Technology) and β-actin (1:2000, Beijing Zhongshan Jinqiao Biological Technology Co. China). Subsequently, the membranes were incubated with HRP-conjugated goat anti-rabbit secondary antibody (1:3000, Beijing Zhongshan Jinqiao Biological Technology Co., China) for 1.5 h at room temperature. The specific protein bands were detected using enhanced chemiluminescence (ECL) Western blotting detection kit (Millipore), and the immunoreactive bands were digitally scanned and analyzed using Image J software. The band intensity of the target proteins was normalized against β-actin that was used as a loading control.

### IHC

The paraffin-embedded brain sections were measured for Bax and Bcl-2 protein expression levels using ChemMate™ Envision + HRP/DAB Rabbit/Mouse Detection Kit (Gene Technology Shanghai Co., China) according to the manufacturer’s instructions. Briefly, coronal sections were deparaffinized and rehydrated. Antigen retrieval was performed by boiling the sections in 10 mM citrate buffer (pH 6.0) for 45 min. After washing with phosphate-buffered saline (PBS), the sections were incubated with 3% H_2_O_2_ in PBS in the dark for 15 min, followed by incubation with the following primary antibodies at 4 °C overnight: anti-Bcl-2 (1:200, Abcam) and anti- Bax (1:200, Abcam). Then, the sections were then incubated with ChemMate™ Envision + HRP for 45 min at room temperature, stained with 3,3-diaminobenzidine (DAB), and counterstained with hematoxylin. For negative controls, additional sections were treated similarly, albeit the primary antibody was omitted. Cells were defined as positive if the cytoplasm was stained brown. Images were obtained for five view fields per section at 400× magnification using an Olympus microscope. The integrated optical density (IOD) and the area of positively-stained tissues were obtained using Image-Pro Plus 6.0 software. The mean density (IOD/area) was calculated and averaged.

### Statistical analysis

Data was expressed as mean ± standard deviation. Differences between the groups were analyzed by one-way ANOVA or Student’s t-test. The counting differences were analyzed by Fisher’s exact test. P < 0.05 was considered statistically significant.

## Abbreviations

HIE: hypoxic-ischemic encephalopathy
EPO: erythropoietin
rhEPO: recombinant human erythropoietin
CEPO: carbamylated erythropoietin
CNS: central nervous system
EPOR: erythropoietin receptor
BBB: blood–brain barrier
Lys-C: carboxyl side of lysine
HI: ischemia–hypoxia
I/R: hypoxic-ischemic reperfusion
CC3: cleaved caspase-3
IHC: immunohistochemistry
Bcl-2: B cell lymphoma
Bax: Bcl-2 Associated X Protein
SD: Sprague–Dawley
i.p.: intraperitoneal
KOCN: potassium cyanate
DTE: dithiothreitol
HE: hematoxylin–eosin
SDS-PAGE: sodium dodecyl sulfate-polyacrylamide gel electrophoresis
TUNEL: terminal deoxynucleotidyl transferase-mediated dUTP nick end-labeling
TdT: terminal deoxynucleotidyl transferase
RIPA: radioimmunoprecipitation
PMSF: phenylmethylsulfonylfluoride
PVDF: polyvinylidene difluoride
TBST: tween 20
ECL: chemiluminescence
PBS: phosphate-buffered saline
DAB: 3,3-diaminobenzidine
IOD: integrated optical density
